# Hemispheric differences in the mesostriatal dopaminergic system

**DOI:** 10.3389/fnsys.2014.00110

**Published:** 2014-06-11

**Authors:** Ilana Molochnikov, Dana Cohen

**Affiliations:** The Leslie and Susan Gonda Multidisciplinary Brain Research Center, Bar-Ilan UniversityRamat-Gan, Israel

**Keywords:** dopamine, laterality, striatum, side preference, VTA, nucleus accumbens

## Abstract

The mesostriatal dopaminergic system, which comprises the mesolimbic and the nigrostriatal pathways, plays a major role in neural processing underlying motor and limbic functions. Multiple reports suggest that these processes are influenced by hemispheric differences in striatal dopamine (DA) levels, DA turnover and its receptor activity. Here, we review studies which measured the concentration of DA and its metabolites to examine the relationship between DA imbalance and animal behavior under different conditions. Specifically, we assess evidence in support of endogenous, inter-hemispheric DA imbalance; determine whether the known anatomy provides a suitable substrate for this imbalance; examine the relationship between DA imbalance and animal behavior; and characterize the symmetry of the observed inter-hemispheric laterality in the nigrostriatal and the mesolimbic DA systems. We conclude that many studies provide supporting evidence for the occurrence of experience-dependent endogenous DA imbalance which is controlled by a dedicated regulatory/compensatory mechanism. Additionally, it seems that the link between DA imbalance and animal behavior is better characterized in the nigrostriatal than in the mesolimbic system. Nonetheless, a variety of brain and behavioral manipulations demonstrate that the nigrostriatal system displays symmetrical laterality whereas the mesolimbic system displays asymmetrical laterality which supports hemispheric specialization in rodents. The reciprocity of the relationship between DA imbalance and animal behavior (i.e., the capacity of animal training to alter DA imbalance for prolonged time periods) remains controversial, however, if confirmed, it may provide a valuable non-invasive therapeutic means for treating abnormal DA imbalance.

## Introduction

There is a general consensus regarding cerebral dominance and hemispheric specialization in the human brain in which the right hemisphere is dominant for analyzing complex visuo-spatial relationships and for the perception and expression of emotions, whereas language is represented in the left hemisphere (Kimura and Archibald, 1974; Gazzaniga, 1995; Davidson and Kenneth, 2004; Fox and Reed, 2008; Gotts et al., 2013). Healthy subjects display asymmetries in the DA system, which in humans have been associated, for example, with lateralized motor performance and hand preference (Bracha et al., 1987; De La Fuente-Fernandez et al., 2000; Mohr and Bracha, 2004). Pathological DA asymmetries occur in neuropsychiatric and neurodegenerative disorders such as schizophrenia, depression, and Parkinson's disease (Peterson et al., 1993; Seibyl et al., 1995; Gruzelier, 1999; Hietala et al., 1999; Van Dyck et al., 2002; Hsiao et al., 2003, 2013). Inter-hemispheric imbalance in DA concentration and its metabolites has been described in the rodent mesostriatal system and in related structures such as prefrontal cortex (PFC), Entorhinal cortex (EC) and the hippocampus (Glick et al., 1982; Schneider et al., 1982; Denenberg and Rosen, 1983; Drew et al., 1986; Fride and Weinstock, 1987; Nowak, 1989; Rodriguez et al., 1994; Becker, 1999; Hietala et al., 1999; Thiel and Schwarting, 2001; Van Dyck et al., 2002; Silva et al., 2007; Vernaleken et al., 2007; Cannon et al., 2009; Martin-Soelch et al., 2011; Hsiao et al., 2013). This DA imbalance in the mesostriatal system has been associated with a variety of motor and cognitive aspects of behavior among which are spatial performance such as side preference in a T maze or during lever-press (Zimmerberg et al., 1974; Glick et al., 1981; Castellano et al., 1987), locomotor activity (Glick and Ross, 1981; Glick et al., 1988; Cabib et al., 1995; Nielsen et al., 1997; Budilin et al., 2008), difference in sensitivity to intracranial self-stimulation, motivation, responsiveness to stress, and appetitive and aversive stimuli (Glick et al., 1981; Carlson et al., 1991, 1993; Besson and Louilot, 1995; Sullivan and Gratton, 1998; Berridge et al., 1999; Sullivan and Dufresne, 2006; Fox and Reed, 2008; Tomer et al., 2008, 2014; Laplante et al., 2013).

In this review, we address the midbrain DA system functionality by examining the laterality of the mesostriatal system in populations of animals as reflected in DA imbalance across hemispheres. To do so, we specify the type, origin and destination of primarily midbrain DA neurons, review evidence supporting DA related hemispheric laterality as assessed by measuring DA levels and its metabolite dihydroxyphenylacetic acid (DOPAC), examine correlations between DA imbalance and spatial and cognitive biases in animal behavior, and finally, we discuss the ways in which these correlations are altered in response to unilateral and systemic biochemical intervention and behavioral manipulation. We particularly ask whether or not manipulations generate a symmetrical effect across hemispheres (i.e., whether a unilaterally applied manipulation generates a mirror image of the effect generated by applying that manipulation to the other hemisphere). The search for papers was done using relevant key words such as asymmetry, laterality, DA, ventral tegmental area (VTA), substantia nigra (SN), striatum, nucleus accumbens (NAc), PFC, medial forebrain bundle (MFB), 6-hydroxydopamine (6-OHDA), unilateral, left, right, inter hemispheric, mesostriatal, mesolimbic, nigrostriatal, affective, limbic, etc. Papers were selected irrespective of results if reported data comprised left/right information without collapsing them across hemispheres.

## Anatomy and connectivity

The two prime dopaminergic fiber groups, the A9 and A10, are located in the ventral midbrain (Dahlstroem and Fuxe, 1964). The A10 group is the largest group of DAergic cells in the ventral midbrain tegmentum which is located for the most part in the VTA. The A10 group represents about two thirds (~65%) of VTA cell population. In addition, the VTA comprises a sizable population of GABAergic inhibitory neurons (~30%) and excitatory glutamatergic neurons (~5%) (Nauta et al., 1978; Johnson and North, 1992). This DAergic group gives rise to the mesolimbic DA system projecting through the MFB primarily to the ventral striatum (NAc) (Dahlstroem and Fuxe, 1964). The A9 group is the most densely packed group of DAergic cells, which is located in the substantia nigra pars compacta (SNc) and accounts for about 90% of the neuronal population in this relatively homogenous structure, in addition to a small population of GABAergic inhibitory neurons (10%). The A9 group constitutes the nigrostriatal DAergic pathway which projects through the MFB to the dorsal striatum (dStr) composed of the caudate and putamen. Another small group of DAergic cells in rodents and primates is the A8 group, located in the midbrain reticular formation.

The DAergic signal arising from these midbrain structures enables basal ganglia (BG) control of motor planning and action selection (Wurtz and Hikosaka, 1986; Berns and Sejnowski, 1998; Gurney et al., 2001) by two parallel pathways diverging in the striatum and separating the BG loop into the direct, striatonigral pathway, and the indirect, striatopallidal pathway, via medium-sized spiny projection neurons (MSNs) expressing D1 receptors and D2 receptors, respectively (Alexander and Crutcher, 1990; Gerfen et al., 1990). The subthalamic nucleus which is a part of the indirect pathway also receives direct cortical input via the hyper-direct fast pathway that bypasses the striatum and is therefore independent of striatal DA input (Kita, 1992; Nambu et al., 2000, 2002). The balance between the direct and indirect pathways is thought to be essential for proper BG function (Albin et al., 1989; Wichmann and Delong, 1996; Delong and Wichmann, 2007). The MSNs, which are GABAergic neurons, make up the vast majority (90–95%) of striatal neurons in rodents and primates (Kemp and Powell, 1971; Mensah and Deadwyler, 1974; Wilson and Groves, 1980; Graveland and Difiglia, 1985). The MSNs relay information via the direct and indirect pathways to the two major output stations of the BG, the substantia nigra pars reticulata (SNr) and the globus pallidus internal segment (or the entopeduncular nucleus in rodents) which are both primarily GABAergic (Van Der Kooy and Wise, 1980; Oertel and Mugnaini, 1984; Bolam et al., 1985, 1993; Parent and Hazrati, 1995).

### Connectivity within the mesostriatal systems

The DA neurons in the SNc project predominantly to the striatum, which also receives a small percentage of DA inputs from the SNr and VTA (Van Der Kooy and Wise, 1980). Overall, at least 85% of the fibers reaching the striatum from the midbrain arise from the DA mesostriatal neurons. Of the 15% of the non-DA carrying fibers reaching the striatum, about 2% arrive from the VTA, 3–4% from the SNc, and 8–9% arrive from the SNr (Van Der Kooy et al., 1981; Swanson, 1982; Fallon et al., 1983; Gerfen et al., 1987). In turn, neurons located in the striatal patches (Gerfen, 1984, 1985) project back to the DAergic neurons in the SNc (Gerfen et al., 1987). The two types of SNc neurons also project onto the NAc and comprise about 38% of its midbrain input (Swanson, 1982). The NAc projections back to the nigral complex are substantially larger than the nigrostriatal fibers it receives (Nauta et al., 1978).

All three types of VTA neurons project onto the NAc and comprise 40% of its midbrain input (Phillipson, 1979; Swanson, 1982). Of these NAc afferents, 85% are primarily DAergic and the remaining 15% arise from GABA or glutamate releasing neurons (Swanson, 1982; Gerfen et al., 1987; Gonzalez-Hernandez et al., 2004, p. 23; Stuber et al., 2012; Ishikawa et al., 2013). In turn, the NAc sends inhibitory projections back to VTA GABAergic neurons including those that innervated it, thus enabling reciprocal information transfer (Nauta et al., 1978; Phillipson, 1979; Kalivas et al., 1993). In addition, the VTA DAergic neurons are inhibited by GABAergic interneurons located in the VTA (Stuber et al., 2012; Van Zessen et al., 2012).

Approximately 1–3% of the DA fibers reaching the striatum come from the contralateral SNc, the VTA and the posterior lateral hypothalamic area (Veening et al., 1980; Gerfen et al., 1982; Swanson, 1982; Altar et al., 1983; Fallon et al., 1983; Consolazione et al., 1985; Lieu and Subramanian, 2012). Less than 10% of the VTA efferents cross the midline to the contralateral side (Swanson, 1982) where they give rise to about 8% of the fibers reaching the NAc and about 1–3% of the fibers reaching the dStr (Carter and Fibiger, 1977; Altar et al., 1983; Douglas et al., 1987). This inter-hemispheric communication does not pass through the corpus callosum (Fass and Butcher, 1981) and instead decussates before the fibers reach the lateral hypothalamic area, close to the VTA (Altar et al., 1983; Douglas et al., 1987). It has been shown that VTA and SN neurons project either ipsilaterally or contralaterally but not bilaterally (Loughlin and Fallon, 1982).

### Connectivity between the mesostriatal systems and related structures

The cortical targets of the SN include the PFC, the motor cortex and the EC (Loughlin and Fallon, 1984). All major regions of the cerebral cortex communicate with the striatum bilaterally, however, with ipsilateral predominance (Veening et al., 1980; Fisher et al., 1986; McGeorge and Faull, 1989; Brog et al., 1993; Totterdell and Meredith, 1997; Alloway et al., 2006; Lieu and Subramanian, 2012). Importantly, a single SN neuron sends collaterals to limbic, striatal, and cortical structures, unlike VTA neurons that can project solely to one terminal field (Loughlin and Fallon, 1984).

Projection neurons in the VTA arrive at the PFC and synapse on pyramidal neurons that project to the contralateral PFC (Carr and Sesack, 2000; Hnasko et al., 2012; Stuber et al., 2012) suggesting that the PFC is ideally suited to modulate inter-hemispheric information flow between itself and the BG. Additionally, the PFC sends excitatory glutamatergic projections onto VTA GABAergic neurons which innervate the NAc, and also onto VTA DAergic neurons which project back to the PFC (Carr and Sesack, 2000).

The VTA neurons also project to the EC of which 46% of the projections are DAergic. The EC in turn sends glutamatergic projections to the striatum, mainly to the NAc core (Krayniak et al., 1981; Finch et al., 1995; Moser et al., 2010). Similar to the VTA-NAc connectivity, the majority of the fibers (92%) arrive at the ipsilateral EC whereas the remaining fibers reach the contralateral EC (Swanson, 1982). In addition to the bidirectional communication between the VTA and the NAc, and the VTA-EC-NAc circuit, these structures are also part of a complex loop engaging the hippocampus. Specifically, DAergic neurons in the VTA project to the hippocampus which through the subiculum, NAc and the ventral pallidum releases DA neurons in the VTA from tonic inhibitory ventral pallidum influence (Lisman and Grace, 2005). In addition, the glutamatergic neurons in the VTA project back to the ventral pallidum (Hnasko et al., 2012).

**Figure 1:**
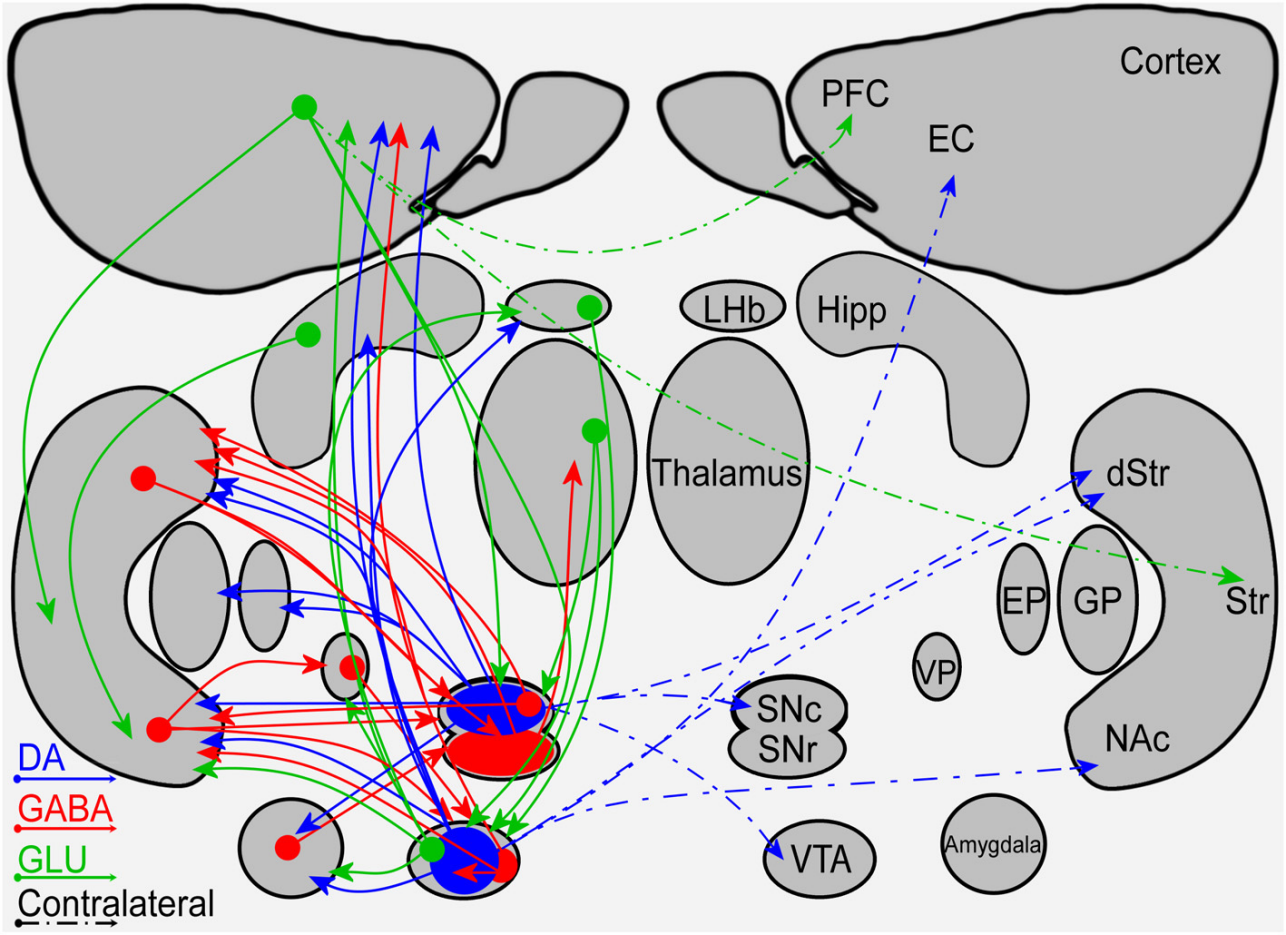
**A schematic diagram of the connectivity within and between the mesostriatal pathways and related structures**. Shown are the ipsilateral (solid lines) and contralateral (dashed lines) projections of fibers containing DA (blue), GABA (red), or glutamate (green). Abbreviations: PFC, Prefrontal Cortex; EC, Entorhinal cortex; Hipp, Hippocampus; LHb, Lateral Habenula; Str, Striatum; dStr, Dorsal Striatum; NAc, Nucleus Accumbens; GP, Globus pallidus; EP, Entopeduncular nucleus; VP, Ventral Pallidum; SNc, Substantia Nigra Pars Compacta; SNr, Substantia Nigra Pars Reticulata; VTA, Ventral Tegmental Area.

Observation of the mesostriatal and mesolimbic DA pathways reveals a symmetrical connectivity with a primary influence in the ipsilateral side and a minor influence in the contralateral side (illustrated in Figure 1). Symmetrical fiber connectivity entails that unilateral manipulations of the DA system will affect mainly the manipulated side and that the outcome of left vs. right manipulations will be symmetrical as long as physiological factors are not taken into account. Inter-hemispheric communication allowing bilateral influence of unilateral manipulation is supported primarily by the PFC without breaking the symmetry. It is noteworthy that information is lacking regarding fiber distribution differences in the left vs. right hemispheres. If fiber distribution differences exist, they may yield asymmetrical, bi-lateral response to a variety of unilateral manipulations.

## Endogenous asymmetry in the DAergic system

Endogenous differences in the DA system of population of animals were observed in adulthood by many groups. These differences were expressed in DA level and metabolites, D1 and D2 receptor concentration, binding potential of the receptors and DA transporters measured in the neocortex, striatum and NAc. However, reports by different laboratories do not always concur in occurrence site, magnitude and the directionality of the observed imbalance (Glick et al., 1982; Schneider et al., 1982; Denenberg and Rosen, 1983; Drew et al., 1986; Fride and Weinstock, 1987; Nowak, 1989; Rodriguez et al., 1994; Giardino, 1996; Becker, 1999; Thiel and Schwarting, 2001; Van Dyck et al., 2002; Silva et al., 2007; Vernaleken et al., 2007; Cannon et al., 2009; Martin-Soelch et al., 2011; Hsiao et al., 2013). Examination of the results and experimental conditions reveals, in addition to methodology of measurement, differences in animals' age, gender ratio, and in the species and strains of animals used. A clue for reconciling this discrepancy may be found in reports describing an inter-hemispheric regulatory/compensatory process which acts differentially in both hemispheres to restore DAergic balance. Such a process has been suggested to be active during development (Rodriguez et al., 1994; Giardino, 1996; Frohna et al., 1997; Vernaleken et al., 2007) and following tissue damage (Altar et al., 1983; Pritzel et al., 1983; Carlson et al., 1996; Roedter et al., 2001; Blesa et al., 2011). This regulatory/compensatory process which is time-dependent utilizes mechanisms of action such as controlling the ratio of D1/D2 receptors (Joyce, 1991a,b), changing the number of synaptic terminals (Jaeger et al., 1983; Lawler et al., 1995; Meshul et al., 1999; Gittis et al., 2011; Golden et al., 2013), and enhancing DA turnover and release from the available fibers (Ikegami et al., 2006). If the influence of this regulatory process on the expression of DA imbalance is experience dependent the discrepancy across laboratories could be reconciled because the variance in animals' experience is likely to be smaller within a laboratory than across laboratories. Hence, observation of hemispheric chemical imbalance may be obscured by the animal's unique experience and therefore studying DA imbalance in relation to animal behavior, preferably during a well-defined task, may minimize the influence of experience and could potentially reveal a robust effect that is consistent across laboratories.

## Behavioral correlates of endogenous DA asymmetry

**Figure 2:**
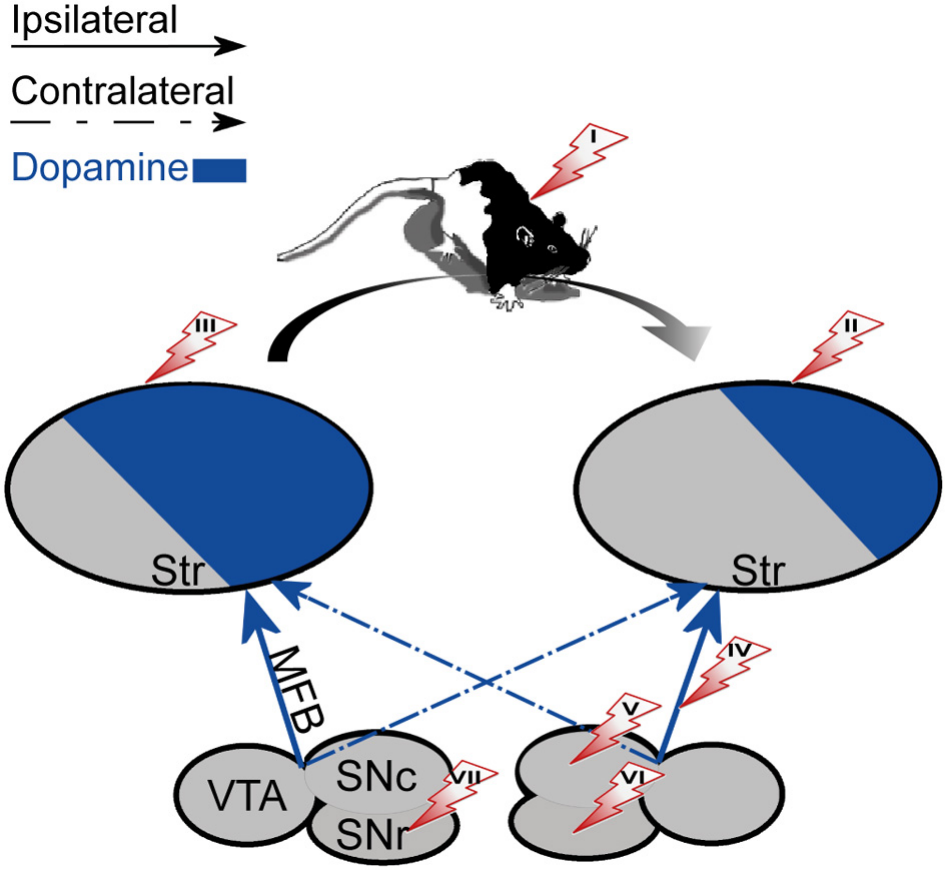
**The effect of unilateral biochemical manipulations**. The specified manipulations were placed on the side that induced rotation in the right direction (marked with an arrow). The direction of rotation was always contralateral to the striatum with a high DA concentration (left). (I) systemic administration of d-amphetamine (enhances natural asymmetry). (II) 6-OHDA lesion of the striatum. (III) DA agonists, Acetylcholine antagonists or NMDA administered to the striatum. (IV) 6-OHDA lesion of the MFB. (V) 6-OHDA lesion or DA administration to the SNc. (VI) GABA antagonists administered to the SNr. (VII) DA agonists, Acetylcholine agonists or GABA agonists administered to the SNr.

The degree of right hand preference in humans positively correlates with left putamen dominance, whereas right caudate dominance positively correlates with the level of performance during simultaneous bimanual movements in right-handed healthy subjects (De La Fuente-Fernandez et al., 2000). Rodents also display paw preference yet this preference is correlated with higher DA and DOPAC concentrations in the NAc ipsilateral to the preferred limb (Cabib et al., 1995; Budilin et al., 2008). Additionally, lateralization of DOPAC/DA ratios in favor of the right ventral striatum was positively related to right-side thigmotaxis (Thiel and Schwarting, 2001). Generally, however, spatial preference in rodents appears to be the manifestation of difference in activity occurring between the nigrostriatal systems. Specifically, rats forced to select a side (left or right) in a T maze displayed significantly higher DA content in the striatum contralateral to the preferred side than to the ipsilateral striatum (Zimmerberg et al., 1974). Similarly, the preferred direction of rotation was directly linked to the asymmetry in baseline DA (see Figure 2): the preferred direction of rotation was contralateral to the striatum with higher DA (Glick et al., 1988). Further support for this observation comes from intracranial self-stimulation experiments showing that the side having a lower threshold for stimulation was contralateral to the direction of spontaneous rotations (Glick et al., 1981). Opposite to DA levels, lower DOPAC values were observed in the striatum contralateral to the preferred direction of rotation (Glick et al., 1977, 1988). However, haloperidol, which decreases the activation of DA receptors, inversed the relation between DOPAC concentration and direction of rotation without changing the animal's preferred direction (Jerussi and Taylor, 1982), suggesting that DOPAC concentration by itself may be a less reliable predictor of rotation direction. The preferred direction of rotation and paw preference in rodents are uncorrelated suggesting that they reflect two distinct processes (Nielsen et al., 1997) each correlated with DA asymmetry in a different striatal sub-region. This distinction in rodents contrasts with data showing a relation between human handedness and preferred direction of rotation; right-handers preferred left-sided turning and non-right-handers preferred right-sided turning (Mohr et al., 2003).

Other brain areas were also linked with spatial preference in rodents. For example, in the two-lever operant conditioning task rats showed a right lever bias that was correlated with enhanced activity in the left frontal cortex (Glick and Ross, 1981). Additionally, enhanced activity in the left PFC was observed during right rotation preference. These findings are consistent with reports of PFC-striatum interactions. Specifically, phasic activation of the PFC has been shown to increase DA release in the ipsilateral NAc (Taber and Fibiger, 1995) probably via glutamate-induced activation of DA neurons in the VTA (Karreman and Moghaddam, 1996).

As opposed to the motor and sensory modalities displaying characteristic laterality, limbic entities such as stressors and natural rewards (food, liquid, or pleasure) lack laterality. Nonetheless, several studies suggest that endogenous DA asymmetry can be correlated with affective behaviors as well. For example, human baseline asymmetry in D2 receptor availability in the left relative to the right striatum was associated with greater positive incentive motivation (Tomer et al., 2008; Martin-Soelch et al., 2011). Moreover, human subjects with higher D2 binding in the left hemisphere were sensitive to learning from positive reinforcement whereas those with higher D2 binding in the right hemisphere were sensitive to learning from negative reinforcement (Tomer et al., 2014). Additionally, right-biased rats were significantly more active and had a stronger side preference than left-biased rats (Glick and Ross, 1981) which also may suggest attention/motivation influence.

## The influence of brain manipulation on animal behavior

In the previous section we reviewed evidence showing correlations between endogenous DA imbalance and natural behavior. In the following section we examine how systemic and unilateral artificial alteration of the inter-hemispheric DA imbalance influences animals' behavior.

### Manipulations in the nigrostriatal system

#### Systemic biochemical manipulations

Systemic application of d-amphetamine, which enhances DA concentration at the DAergic terminals was found to enhance the rate of rotations in the preferred direction prior to the injection (Jerussi and Glick, 1976), as well as the inter-hemispheric difference in striatal DA content in a dose dependent manner (Glick et al., 1981). Moreover, d-amphetamine application further lowered the threshold for MFB activation and consequently further biased the side preference of self-stimulation (Glick et al., 1981). Apomorphine, which is a non-selective DA agonist, induced rotational behavior in rats similar to d-amphetamine. The induced rotation increased with apomorphine dosage until reaching a plateau (Jerussi and Glick, 1975). Conversely, apomorphine administration significantly decreased the number of times rats chose their preferred arm in a T-Maze but did not alter the side preference observed prior to drug application (Castellano et al., 1987) which may indicate drug spread into limbic regions. Inhibition of catecholamine synthesis by Alpha-methyl-para-tyrosine (AMPT) markedly reduced or completely abolished d-amphetamine induced rotation (Jerussi and Glick, 1976), but did not affect rotation elicited by apomorphine (Jerussi and Glick, 1975), suggesting that these two drugs operate differently. Haloperidol prevented the rotation elicited by both d-amphetamine and apomorphine (Jerussi and Glick, 1976), indicating its robust influence.

#### Local biochemical manipulations

A more selective way to induce DA imbalance in the nigrostriatal pathway is by unilaterally injecting DA agonists and antagonists into the dStr or the SN. Figure 2 summarizes the results obtained following unilateral manipulations. Local injection of DA into the SNc induced weak ipsiversive or mixed ipsiversive and contraversive rotation probably due to DA autoreceptors which locally inhibit DA neurons (Jang et al., 2011). Injection of DA into the SNr only induced contraversive circling (Kelly et al., 1984). Local injection of apomorphine to the SNr or SNc had a mild influence on the tendency of the rats to rotate (Kelly et al., 1984), suggesting activation of other neuronal mechanisms that counteract the influence of apomorphine on DAergic neurons. Unilateral injections of apomorphine into the dStr induced contraversive turning as did injections of d-amphetamine, NMDA (Ossowska and Wolfarth, 1995), and atropine which is a muscarinic acetylcholine receptor antagonist (Jerussi and Glick, 1976).

GABA-related drugs and GABA antagonists applied intranigrally also facilitated rotational behavior; GABA, GABA_A_ agonist (e.g., muscimol) and GABA_B_ agonists (e.g., γ-hydroxybutyric acid and baclofen) injected unilaterally into the SNr of rats elicited contraversive turning by disinhibiting the SNr influence on the striatum and enhancing DA activation ipsilaterally to the injection site, whereas unilateral injections of GABA_A_ antagonist (e.g., bicuculline) produced ipsiversive turning by enhancing SNr inhibition of the striatum (Olpe et al., 1977; Scheel-Kruger et al., 1977).

Unilateral optical stimulation of MSNs in the direct or the indirect striatal pathways concurred with previously described manipulations which unilaterally enhanced or attenuated DA level; direct pathway activation led to contraversive rotation, whereas indirect pathway activation yielded ipsiversive rotation (Kravitz et al., 2010). Such a selective activation of the two pathways validates the influence of DA hemispheric imbalance on preferred rotation direction in the absence of a compensatory mechanism influence.

#### Unilateral lesions using 6-hydroxydopamine

6-hydroxydopamine (6-OHDA) is a neurotoxic compound that selectively destroys catecholamine neurons by penetrating their membrane via the DA or noradrenaline reuptake transporters (Simola et al., 2007). The 6-OHDA injection site determines the extent of damage caused by the neurotoxin and its specificity. Unilateral injection in the MFB has been shown to lesion both the uncrossed and crossed projections of the A9 and A10 cell groups converging on the ipsilateral dStr, and produces extensive catecholamine neuron destruction of about 97% of the cells primarily in the ipsilateral SNc and VTA. The contralateral SNc and VTA are less affected by the neurotoxin injection (Altar et al., 1983) because only a small percentage of their efferents (1% and 8% in the SNc and VTA, respectively) pass through the injection site (Iwamoto et al., 1976; Altar et al., 1983; Torres and Dunnett, 2012).

A more specific lesion can be produced by injecting the neurotoxin into the SNc, which leads to the destruction of only the nigrostriatal (A9) pathway, and more focal damage can be created by lesioning sub-regions of the dStr complex, which induces cell death of 50–99% of the SNc neurons depending on the affected dStr volume (reviewed in Deumens et al., 2002). These three procedures serve as classic animal models for Parkinson's disease (Ungerstedt and Arbuthnott, 1970; Simola et al., 2007). All of these lesions produce a biochemical imbalance in which lower DA levels are measured in the ipsilateral-to-lesion dStr than in the contralateral dStr. This biochemical imbalance is correlated with behavioral alterations such as the development of motor slowness and a turning preference bias toward the side of the lesion (Roedter et al., 2001; Simola et al., 2007). Specifically, 6-OHDA lesions of either the SNc, MFB, or the dStr induce ipsiversive-to-lesion rotation (Jerussi and Glick, 1976; Glick et al., 1977; Carman et al., 1991; Annett et al., 1992; Deumens et al., 2002). The lesion-induced turning behavior ceases within the first post-operative week (Pritzel et al., 1983) thus setting an upper bound on the time required for the compensatory processes to become evident.

It is interesting to note that unilateral optical activation of MSNs in the direct/indirect pathway induced contraversive/ipsiversive rotations similar to that induced by 6-OHDA lesions. This finding suggests that alterations in DA concentrations influence the balance in activation of the direct vs. indirect pathways; reduced DA concentration tips the scale toward activation of the striatopallidal pathway whereas enhanced DA concentration emphasizes the striatonigral pathway.

#### Unilateral lesions and systemic biochemical manipulations

Systemic administration of pharmacological agents influenced the direction of turning evoked by the 6-OHDA lesion by enhancing or reducing the lesion-induced biochemical imbalance. d-amphetamine produced ipsilateral circling behavior whereas haloperidol and pimozide, which decrease the activation of DA receptors, produced contralateral circling behavior in rats with 6-OHDA lesion in the SNc (Iwamoto et al., 1976). Electrolytic lesions of the SNc induced a rotation preference similar to that induced by 6-OHDA (Arbuthnott and Crow, 1971; Iwamoto et al., 1976). Systemic administration of apomorphine generated a more complex outcome depending on the type and extent of the resulting tissue damage; apomorphine yielded contraversive circling following 6-OHDA lesions (Iwamoto et al., 1976; Glick et al., 1977; Meshul and Allen, 2000) and the number of rotations depended on the extent of tissue damage. By contrast, apomorphine led to ipsiversive circling following electrolytic lesion (Iwamoto et al., 1976).

Overall, artificial enhancement of striatal DA imbalance in rodents produces circling behavior in the direction toward the side with depleted DA concentration (see Figure 2). The outcome of this category of manipulations which ultimately influence striatal DA processing and transmission is consistent regardless of which component in the circuit has been manipulated and whether the manipulation enhanced or attenuated activity.

### Manipulations in the mesolimbic system

Application of 6-OHDA to the mesolimbic system produced characteristic behavioral deficits different in nature from those produced by lesioning the nigrostriatal system. Unilateral injection of 6-OHDA to the right VTA or SN impaired acquisition of operant tasks (Hritcu et al., 2008). Bilateral small 6-OHDA lesions of the VTA produced a significant increase in spontaneous locomotor activity whereas large 6-OHDA lesions of the VTA or the NAc produced hypoactivity in the open field, a complete blockade of the locomotor stimulating effects of d-amphetamine, and a profound supersensitive response to apomorphine expressed as enhanced locomotion (Koob et al., 1981). Interestingly, a radiofrequency-VTA lesion caused a greater increase in spontaneous activity relative to the VTA 6-OHDA lesion, suggesting the presence of a powerful inhibitory influence of the mesolimbic DA system within the VTA (Koob et al., 1981).

#### Manipulations of structures interacting with the mesolimbic system

Experiments described thus far support the view that the relationship between the manipulation-induced DA imbalance and the animal's induced rotation direction is symmetric across hemispheres (see Figure 2). Experiments directly assessing the existence and function of DA imbalance in the mesolimbic pathway are lacking. However, some of the information can be deduced from experiments addressing the issue indirectly, for example, by applying pharmacological intervention in structures which interact with the mesolimbic system such as the PFC, EC, and hippocampus. Such experiments reveal that despite the apparent anatomical symmetry of the fibers unilateral manipulations influence the DA system asymmetrically. The results of these experiments are summarized in Table 1.

**Table 1:**
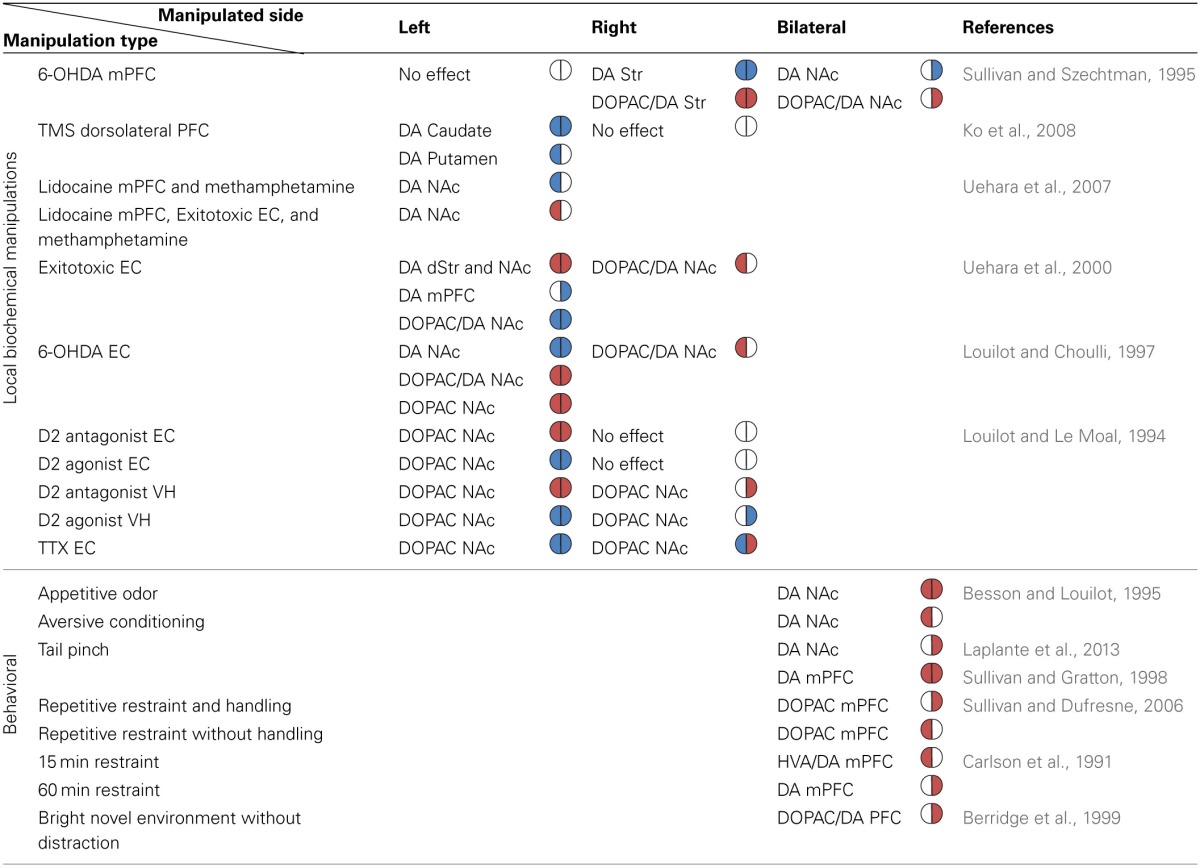
**The reported directionality of DA imbalance in the mesolimbic system following biochemical and behavioral manipulations**.

*A circle denotes the spatial distribution of the manipulation outcome. Colored area indicates a significant increase (red) or decrease (blue) in DA or its metabolites in the corresponding hemisphere. Data were included from different species, ages, and measurement techniques*.

#### The effect of PFC manipulations on the mesolimbic DA system

The PFC represents information in the short term working memory (Kolb et al., 1994; Kesner et al., 1996; Goldman-Rakic, 2011) and is crucial for spatial and emotional response selection which are influenced by mesocortical DA (Thierry et al., 1976; Delatour and Gisquet-Verrier, 1999; Sullivan, 2004). As previously mentioned the interaction between mesostriatal DA and PFC is reciprocal (Carr and Sesack, 2000; Hnasko et al., 2012; Stuber et al., 2012). 6-OHDA lesions of the mesocortical DA innervating the right mPFC and anterior cingulate induced a significant bilateral reduction in DA content and an increase in DA turnover in the striatum (Sullivan and Szechtman, 1995). Similar lesions to the left mPFC and anterior cingulate did not affect striatal DA. Moreover, similar to unilateral lesion of the right PFC, bilateral lesions also reduced DA content and increased DA turnover but the response was limited to the right NAc (Sullivan and Szechtman, 1995). Evidence for the asymmetrical influence exerted by the PFC on the striatum also comes from studies performed on human subjects. For example, application of a continuous TMS theta burst stimulation (cTBS) to the left dorsolateral PFC of healthy young right-handed adults inhibited DA release bilaterally in the caudate and ipsilaterally in the putamen, whereas right dorsolateral PFC cTBS did not influence binding potential in the striatum (Ko et al., 2008).

A few studies correlated DA imbalance following chemical manipulations of the PFC with behavior. For example, correlations were observed between DA depletion induction in the right medial PFC (mPFC) and rats exhibiting the most severe stress-induced gastric pathology—ulcers (Sullivan and Szechtman, 1995). Additionally, unilateral injection of DA (D1/D2) antagonist into the mPFC significantly enhanced the restraint stress-induced increases in corticotrophin and corticosterone in non-handled rats (Sullivan and Dufresne, 2006). However, similar experiments in handled rats showed that only right side DA receptor blockade induced elevation of peak stress hormone levels (Sullivan and Dufresne, 2006). These data provide supporting evidence for the asymmetrical relationship between DA imbalance in the mesolimbic system and the animals' affective behavior, and further emphasize that the animals' experience may hamper attempts to characterize this relationship.

#### The effect of EC and hippocampus manipulations on the mesolimbic DA system

The EC and the ventral hippocampus (VH) receive DAergic projections from the VTA, and depending on event novelty (Lisman and Grace, 2005) act upon the VTA to regulate the DAergic transmission to the NAc (Louilot and Le Moal, 1994; Kurachi et al., 2000). Inter-hemispheric comparison of changes in DA and DOPAC concentration in the NAc and related regions following pharmacological intervention in the EC or VH revealed a time-dependent process with complex lateralization. Excitotoxic lesion of the left EC decreased DOPAC levels in the NAc and the mPFC 2 weeks post lesion (Kurachi et al., 2000). Moreover, measurements performed in adolescence following excitotoxic lesion of the left EC in newborn rats showed bilateral enhancement of DA concentration in the NAc and dStr, and a unilateral decrease in the right mPFC. The NAc also displayed a bilateral decrease in DOPAC/DA concentration ratio whereas the dStr did not. None of these alterations in the DA system was detected in the newborns (Uehara et al., 2000). When the left EC of adult rats was lesioned with 6-OHDA the outcome was reversed; DA tissue content decreased whereas DOPAC tissue content and the resultant DOPAC/DA ratio increased in the NAc (Louilot and Choulli, 1997). It remains to be seen whether the differences in outcome are due to the age at which the lesion was made or due to the lesion type. A similar lesion to the right EC caused elevation in DOPAC/DA ratio only in the left NAc (Louilot and Choulli, 1997). A more specific intervention by manipulation of both D1 and D2 receptors in the EC and VH significantly influenced DOPAC concentrations in the NAc such that the effect of the D2 manipulation was more pronounced than that of D1 (Louilot and Le Moal, 1994). Specifically, administration of a D2 receptor antagonist in the left EC increased DOPAC concentration in both NAc whereas administration of a D2 agonist caused the opposite effect (i.e., decreased DOPAC concentration in both NAc). Injection of a D2 antagonist or agonist into the right EC did not influence DOPAC levels in the NAc. Injections of a D2 antagonist and agonist into the VH evoked similar responses as left EC injections but the response was limited to the side ipsilateral to the injected hemisphere (Louilot and Le Moal, 1994). Moreover, injection of the neurotoxic compound Tetrodotoxin (TTX) into the left EC decreased DOPAC in both NAc whereas TTX in the right EC increased DA transmission in the ipsilateral NAc and decreased DOPAC in the contralateral NAc (Louilot and Le Moal, 1994). Unilateral TTX injections into the VH decreased DOPAC in the NAc of both hemispheres irrespective of injection side (Louilot and Le Moal, 1994). These side-dependent alterations in NAc DA release and its metabolites support the existence of an asymmetrical functional interdependence between midbrain DAergic pathways reaching the EC and the NAc (Louilot and Choulli, 1997).

Consistent with altered mesolimbic DAergic transmission, a left EC excitotoxic lesion in adult rats enhanced spontaneous and methamphetamine induced locomotor activity (Sumiyoshi et al., 2004; Kopniczky et al., 2006). Interestingly, the methamphetamine-induced normalized DA release in the ipsilateral NAc or dStr did not change in comparison to sham-operated animals possibly due to enhanced postsynaptic sensitivity rather than presynaptic alteration in DA levels (Uehara et al., 2000; Sumiyoshi et al., 2004). When the excitotoxic lesion to the left EC was combined with ipsilateral inactivation of the mPFC by lidocaine (a non-selective blocker of axonal fibers of passage as well as neurons) the methamphetamine-induced DA release in the ipsilateral NAc was substantially enhanced (Uehara et al., 2007). By contrast, ipsilateral inactivation of the mPFC by lidocaine alone reduced the methamphetamine-induced DA release in the NAc, which is consistent with previous data. Overall, inactivation of the mPFC together with structural abnormalities in the EC leads to deregulation of DAergic neurotransmission in limbic regions (Uehara et al., 2007).

The results described thus far strengthen assumptions concerning the lateralized involvement of the PFC, the EC, and the VH in the modulation and possibly regulation of mesostriatal DA asymmetry (Glick and Ross, 1981; Glick and Carlson, 1989; Fox and Reed, 2008). Overall, it seems that the nigrostriatal system displays symmetrical laterality (i.e., the outcome of different manipulations applied to one hemisphere is a mirror image of the outcome obtained when the manipulation is applied to other hemispheres), whereas the DA mesolimbic system and related structures display asymmetrical laterality (i.e., the outcome of different unilateral manipulations is not a mirror image of the same manipulation applied to the other hemisphere), The occurrence of an asymmetrical laterality supports the notion of specialized hemispheric function (Sullivan, 2004; Fox and Reed, 2008).

## The influence of animal training on DA imbalance in the nigrostriatal system

Thus, far we have described how endogenous DA imbalance in the nigrostriatal system correlates with animal behavior and how accentuation of this biochemical imbalance can alter behavior. Below we address the opposite question of whether utilizing different behavioral tasks can induce or influence the basal DA imbalance.

Prior to training on an electrified T-maze (Zimmerberg et al., 1974) a relatively small percentage (about 54–59%) of animals displayed side preference (Castellano et al., 1987). After training the vast majority (85.71%) of the rats displayed a side preference with a strong bias toward the right arm (80%—right arm preference; 20%—left arm preference) (Castellano et al., 1987). Unilateral lesions using 6-OHDA ipsilaterally to the preferred side did not change preference parameters, whereas contralateral lesion massively and persistently decreased the choice of the side preferred preoperatively (Castellano et al., 1987). A similar outcome was observed when rats were trained to circle for a sucrose reward in a randomly assigned direction. Prior to training, caudate and NAc DA and DOPAC levels were the same in both hemispheres. By contrast, following training a significant increase occurred in both DA and DOPAC concentrations in the caudate and NAc contralateral to the turning direction whereas these concentrations did not change in the ipsilateral caudate compared to control animals (Yamamoto and Freed, 1982, 1984a). Amphetamine administration further enhanced turning in the trained direction regardless of the animals' previous circling preference. As in naive animals, amphetamine-induced circling led to increased DA concentrations in the caudate contralateral to the trained circling direction (Yamamoto and Freed, 1984b). These findings are congruent with the previously described relationship between DA imbalance and side preference.

Similar experiments (Szostak et al., 1986, 1988, 1989; Glick and Carlson, 1989) in which water-deprived animals were trained on circling behavior failed to replicate the results described by Yamamoto et al. (Yamamoto and Freed, 1982, 1984a,b). In particular, circling did not produce a biochemical imbalance in the nigrostriatal and the mesolimbic DA system; rats trained to circle using a continuous schedule of reinforcement did not exhibit any change in concentrations of striatal DA or DOPAC although alterations in DA and DA turnover were detected in the mPFC (Szostak et al., 1986; Glick and Carlson, 1989). Changing the reward schedule induced enhanced DAergic activity in both the NAc and the dStr (Szostak et al., 1986). A plausible explanation arises from a report which showed that different regions of the striatum exhibit different DA and DOPAC concentrations, thus suggesting striatal region specialization (Szostak et al., 1989). Importantly, the use of a water deprivation protocol without circling training was sufficient to induce a bilateral decrease in DOPAC/DA ratio in the NAc and mPFC (Glick and Carlson, 1989).

The following experiments further strengthen the relationship between DA imbalance and rotation direction. Unilateral 6-OHDA lesions of the MFB of rats trained to circle in their preferred direction for water reinforcement revealed a complex scenario that fits relatively well with the chemical imbalance described in control animals. Specifically, rats with a lesion contralateral to their trained direction stopped turning in that direction and often turned in the untrained direction. By contrast, rats lesioned ipsilaterally to the direction of reinforced circling exhibited only a 50% decrease in the rate of reinforced responding. These findings also highlight the effect of training on DA hemispheric imbalance. Following these procedures the experimental contingencies were reversed such that all rats had to turn in the untrained direction. After the reversal, the contralaterally lesioned group had to turn toward the lesion and consequently easily acquired the reversal, however, reinforced rates of responding did not reach preoperative rates possibly due to altered motivation (Mogenson et al., 1980; Smith et al., 2002; Wise, 2002; Solstad et al., 2008). Conversely, the ipsilaterally lesioned group had to turn away from the lesion and consequently was unable to acquire the reversal and continued to turn in the originally trained direction (Szostak et al., 1988). These findings are in line with the previously described rotation direction induced by 6-OHDA lesions. Moreover, these experiments suggest a causal relationship between DA imbalance and rotation direction because they imply that the direction of circling cannot be altered by training as long as the biochemical imbalance cannot be reversed.

Overall, by considering that alterations in DA/DOPAC concentrations resulting from training appear to be sensitive to variations in the training protocol such as reward schedule, severity and type of deprivation (food or water), type of reward delivered (sucrose, water, or food) and level of performance reached following training, the question whether DA imbalance may be effectively manipulated by training, which is a simple non-invasive procedure and as such may have a therapeutic capacity, remains controversial. These findings also raise the question whether the observed behavioral bias is a byproduct of the current DA imbalance or whether the regulatory mechanism promotes actions aimed to attenuate the inherent hemispheric laterality by overworking the less active regions.

## The influence of animal training on DA imbalance in the mesolimbic system

The question whether behavioral manipulations can induce or influence DA imbalance in the mesolimbic system has been addressed by utilizing different behavioral paradigms involving stress. Stress induction lacks laterality and therefore in all of the experiments emergent results reflect internal network properties. In this section we focus primarily on experiments showing DA measures in the NAc and PFC in attempt to identify whether laterality appears. A summary of the results is shown in Table 1.

DA responses to a naturally attractive olfactory stimulus were elevated in both NAc, most markedly in the right core. However, conditioned taste aversion following LiCl treatment resulted in elevation in DA levels in the left but not right NAc core which further expanded to the left shell upon a second presentation of the now aversive stimulus (Besson and Louilot, 1995). Different results were obtained when instead of presenting aversive stimulus physical stress was applied. The amplitude of DA release following tail pinch was higher in the right NAc compared to the left (Laplante et al., 2013) whereas in the mPFC DA release increased bilaterally (Sullivan and Gratton, 1998). Moreover, the duration of DA response in the left mPFC was significantly longer than that in the right mPFC (Sullivan and Gratton, 1998).

DA measures in the right mPFC correlated with reduced stress levels following 5 repetitions of restraint stress induction in both handled and non-handled animals (Sullivan and Dufresne, 2006). However, in handled rats DA turnover in the right mPFC was significantly higher than the left whereas in non-handled rats DA turnover in the left mPFC was significantly higher relative to the right mPFC (Sullivan and Dufresne, 2006). It should be noted however, that these results are incongruent with earlier findings regarding the cortical lateralization of emotional regulation which is facilitated by early postnatal handling stimulation, and fails to occur in the absence of postnatal handling (Denenberg, 1981; Sullivan and Dufresne, 2006). DA turnover in the right mPFC was higher than the left also in animals that received a foot shock without being able to terminate it (Carlson et al., 1993). When animals could control foot shock termination, DA turnover increased in the mPFC bilaterally (Carlson et al., 1993). Similarly, when rats and mice were exposed to a brightly lit novel environment (novelty stress) DA turnover in the right PFC was significantly higher than the left PFC in animals that weren't given the option of engaging in a non-escape behavior (e.g., chewing an inedible object), compared to those who did (Berridge et al., 1999). Thus, once animals are able to attenuate the physiological stress by controlling stressor termination or by engaging in a coping or displacement behavior the stress-induced DA response in the right PFC is attenuated (Berridge et al., 1999).

DA concentration measurements following different durations of restraint stress provide additional support for the correlation observed between stress level and DA imbalance in the mPFC. DA concentration increased in the right mPFC and reached significance relative to the left mPFC after the animals were kept restraint for an hour, and DA turnover was significantly higher in the left relative to the right mPFC following 15 min of restraint stress but not following prolonged restraint duration. These experiments suggest an alternative hypothesis in which a left to right shift in the mesocortical DA activation occurs with prolonged exposure to stress (Carlson et al., 1991).

The lack of agreement between these reports suggests that laterality in the mesolimbic DA system depends on factors such as the type of induced stress, stress duration and animal's previous experience (Denenberg, 1981; Fox and Reed, 2008). Therefore, these experiments do not allow addressing the question of whether causality exists between DA imbalance in the mesolimbic system and behavior; whether DA imbalance can reduce stress levels or alternatively that DA imbalance is determined by the animals' sensitivity to stress induction should be addressed by utilizing different experimental methods. In any case, despite the absence of a consistent link between behavioral manipulations lacking directionality and DA imbalance in the mesolimbic system these experiments support an asymmetrical processing characteristic of a specialized left vs. right network.

## Discussion

DA imbalance across brain hemispheres is inherent in newborns. With development, this imbalance decreases due to an inter-hemispheric time-dependent regulatory mechanism which differentially influences the DA system in each hemisphere (Rodriguez et al., 1994; Giardino, 1996; Frohna et al., 1997; Vernaleken et al., 2007). This mechanism also compensates for alterations in the endogenous DA imbalance following brain lesion and possibly neurodegenerative processes. The existence of such a mechanism raises the question whether there is a way to influence DA imbalance by utilizing specially designed behavioral manipulations that directly or indirectly activate the mechanism. Although controversial, studies in the nigrostriatal system suggest this possibility is feasible whereas in the mesolimbic system the feasibility of this approach remains to be explored. If successful, this approach can influence the development of novel non-invasive therapeutic means for the treatment of various disorders affected by alterations in DA imbalance.

The EC, the hippocampus, and PFC are part of a network which modulates NAc responses to DA arriving from the VTA. This network seems to display asymmetric left-right laterality rather than displaying the symmetric laterality observed in the nigrostriatal system. This difference in laterality is consistent with the roles played by each BG circuit: the sensorimotor and associative regions (dStr) display laterality which matches the laterality of their sensory inputs, whereas the limbic regions (NAc) which process abstract inputs supposedly lacking laterality are sensitive to the laterality of prefrontal and temporal lobe structures. Such laterality fits well with the concept of hemispheric specialization described in the PFC in relation to various behaviors (Clark et al., 2003; Sullivan, 2004; Goel et al., 2007; Fox and Reed, 2008; Lupinsky et al., 2010). Additional studies are required to determine whether the observed left-right laterality in the mesolimbic system has functional implications for information processing in subcortical structures (e.g., BG) or does it merely reflect the asymmetric functionality in structures such as the EC and the PFC.

The DA system plays a major role in the planning and execution of movements and in acquisition and expression of learned appetitive behaviors which allow the organism to adapt to its surrounding and thus essential for animals survival. To enable comprehensive understanding of the structure and function of this system, it is essential to plan and execute experiments which in addition to factors such as age, gender, and previous experience, take into account the existence of hemispheric specialization, the endogenous DA imbalance and its influence on behavior, and the way in which behavior can influence this imbalance.

### Conflict of interest statement

The authors declare that the research was conducted in the absence of any commercial or financial relationships that could be construed as a potential conflict of interest.

## References

[B1] Albin R. L. Young A. B. Penney J. B. (1989). The functional anatomy of basal ganglia disorders. Trends Neurosci. 12, 366–375 10.1016/0166-2236(89)90074-X 2479133

[B2] Alexander G. E. Crutcher M. D. (1990). Functional architecture of basal ganglia circuits: neural substrates of parallel processing. Trends Neurosci. 13, 266–271 10.1016/0166-2236(90)90107-L 1695401

[B3] Alloway K. D. Lou L. Nwabueze-Ogbo F. Chakrabarti S. (2006). Topography of cortical projections to the dorsolateral neostriatum in rats: multiple overlapping sensorimotor pathways. J. Comp. Neurol. 499, 33–48 10.1002/cne.21039 16958106

[B4] Altar A. Neve K. A. Loughlin S. E. Marshall J. F. Fallon J. H. (1983). The crossed mesostriatal projection: neurochemistry and developmental response to lesion. Brain Res. 279, 1–8 10.1016/0006-8993(83)90157-9 6416611

[B5] Annett L. E. Rogers D. C. Hernandez T. D. Dunnett S. B. (1992). Behavioural analysis of unilateral monoamine depletion in the marmoset. Brain 115(Pt 3), 825–856 10.1093/brain/115.3.825 1352726

[B6] Arbuthnott G. W. Crow T. J. (1971). Relation of contraversive turning to unilateral release of dopamine from the nigrostriatal pathway in rats. Exp. Neurol. 30, 484–491 10.1016/0014-4886(71)90149-X 5554236

[B7] Becker J. B. (1999). Gender differences in dopaminergic function in striatum and nucleus accumbens. Pharmacol. Biochem. Behav. 64, 803–812 10.1016/S0091-3057(99)00168-9 10593204

[B8] Berns G. S. Sejnowski T. J. (1998). A computational model of how the basal ganglia produce sequences. J. Cogn. Neurosci. 10, 108–121 10.1162/089892998563815 9526086

[B9] Berridge C. W. Mitton E. Clark W. Roth R. H. (1999). Engagement in a non-escape (displacement) behavior elicits a selective and lateralized suppression of frontal cortical dopaminergic utilization in stress. Synapse 32, 187–197 10.1002/(SICI)1098-2396(19990601)32:3&lt;187::AID-SYN5&gt;3.0.CO;2-9 10340629

[B10] Besson C. Louilot A. (1995). Asymmetrical involvement of mesolimbic dopaminergic neurons in affective perception. Neuroscience 68, 963–968 10.1016/0306-4522(95)00255-H 8545002

[B11] Blesa J. Juri C. Garcia-Cabezas M. A. Adanez R. Sanchez-Gonzalez M. A. Cavada C. (2011). Inter-hemispheric asymmetry of nigrostriatal dopaminergic lesion: a possible compensatory mechanism in Parkinson's disease. Front. Syst. Neurosci. 5:92 10.3389/fnsys.2011.00092 22287944 PMC3258666

[B12] Bolam J. P. Powell J. F. Wu J. Y. Smith A. D. (1985). Glutamate decarboxylase-immunoreactive structures in the rat neostriatum: a correlated light and electron microscopic study including a combination of Golgi impregnation with immunocytochemistry. J. Comp. Neurol. 237, 1–20 10.1002/cne.902370102 4044888

[B13] Bolam J. P. Smith Y. Ingham C. A. Von Krosigk M. Smith A. D. (1993). Convergence of synaptic terminals from the striatum and the globus pallidus onto single neurones in the substantia nigra and the entopeduncular nucleus. Prog. Brain Res. 99, 73–88 10.1016/S0079-6123(08)61339-4 7509081

[B14] Bracha H. S. Seitz D. J. Otemaa J. Glick S. D. (1987). Rotational movement (circling) in normal humans: sex difference and relationship to hand, foot and eye preference. Brain Res. 411, 231–235 10.1016/0006-8993(87)91074-2 3607430

[B15] Brog J. S. Salyapongse A. Deutch A. Y. Zahm D. S. (1993). The patterns of afferent innervation of the core and shell in the “accumbens” part of the rat ventral striatum: immunohistochemical detection of retrogradely transported fluoro-gold. J. Comp. Neurol. 338, 255–278 10.1002/cne.903380209 8308171

[B16] Budilin S. Y. Midzyanovskaya I. S. Shchegolevskii N. V. Ioffe M. E. Bazyan A. S. (2008). Asymmetry in dopamine levels in the nucleus accumbens and motor preference in rats. Neurosci. Behav. Physiol. 38, 991–994 10.1007/s11055-008-9082-6 18975098

[B17] Cabib S. D'Amato F. R. Neveu P. J. Deleplanque B. Le Moal M. Puglisi-Allegra S. (1995). Paw preference and brain dopamine asymmetries. Neuroscience 64, 427–432 10.1016/0306-4522(94)00401-P 7700530

[B18] Cannon D. M. Klaver J. M. Peck S. A. Rallis-Voak D. Erickson K. Drevets W. C. (2009). Dopamine type-1 receptor binding in major depressive disorder assessed using positron emission tomography and [11C]NNC-112. Neuropsychopharmacology 34, 1277–1287 10.1038/npp.2008.194 18946469 PMC2656589

[B19] Carlson J. N. Fitzgerald L. W. Keller R. W. Jr. Glick S. D. (1991). Side and region dependent changes in dopamine activation with various durations of restraint stress. Brain Res. 550, 313–318 10.1016/0006-8993(91)91333-V 1884238

[B20] Carlson J. N. Fitzgerald L. W. Keller R. W. Jr. Glick S. D. (1993). Lateralized changes in prefrontal cortical dopamine activity induced by controllable and uncontrollable stress in the rat. Brain Res. 630, 178–187 10.1016/0006-8993(93)90655-7 8118684

[B21] Carlson J. N. Visker K. E. Keller R. W. Jr. Glick S. D. (1996). Left and right 6-hydroxydopamine lesions of the medial prefrontal cortex differentially alter subcortical dopamine utilization and the behavioral response to stress. Brain Res. 711, 1–9 10.1016/0006-8993(95)01290-7 8680850

[B22] Carman L. S. Gage F. H. Shults C. W. (1991). Partial lesion of the substantia nigra: relation between extent of lesion and rotational behavior. Brain Res. 553, 275–283 10.1016/0006-8993(91)90835-J 1681983

[B23] Carr D. B. Sesack S. R. (2000). Projections from the rat prefrontal cortex to the ventral tegmental area: target specificity in the synaptic associations with mesoaccumbens and mesocortical neurons. J. Neurosci. 20, 3864–3873 10804226 10.1523/JNEUROSCI.20-10-03864.2000 PMC6772693

[B24] Carter D. A. Fibiger H. C. (1977). Ascending projections of presumed dopamine-containing neurons in the ventral tegmentum of the rat as demonstrated by horseradish peroxidase. Neuroscience 2, 569–576 10.1016/0306-4522(77)90052-5 917282

[B25] Castellano M. A. Diaz-Palarea M. D. Rodriguez M. Barroso J. (1987). Lateralization in male rats and dopaminergic system: evidence of right-side population bias. Physiol. Behav. 40, 607–612 10.1016/0031-9384(87)90105-3 3671525

[B26] Clark L. Manes F. Antoun N. Sahakian B. J. Robbins T. W. (2003). The contributions of lesion laterality and lesion volume to decision-making impairment following frontal lobe damage. Neuropsychologia 41, 1474–1483 10.1016/S0028-3932(03)00081-2 12849765

[B28] Consolazione A. Bentivoglio M. Goldstein M. Toffano G. (1985). Evidence for crossed catecholaminergic nigrostriatal projections by combining wheat germ agglutinin-horseradish peroxidase retrograde transport and tyrosine hydroxylase immunocytochemistry. Brain Res. 338, 140–143 10.1016/0006-8993(85)90256-2 2862949

[B29] Dahlstroem A. Fuxe K. (1964). Evidence for the existence of monoamine-containing neurons in the central nervous system. I. Demonstration of monoamines in the cell bodies of brain stem neurons. Acta Physiol. Scand. Suppl. Suppl. 232, 231–255 14229500

[B30] Davidson R. J. Kenneth H. (2004). The Asymmetrical Brain. Cambridge, MA: MIT Press

[B31] De La Fuente-Fernandez R. Kishore A. Calne D. B. Ruth T. J. Stoessl A. J. (2000). Nigrostriatal dopamine system and motor lateralization. Behav. Brain Res. 112, 63–68 10.1016/S0166-4328(00)00165-0 10862936

[B32] Delatour B. Gisquet-Verrier P. (1999). Lesions of the prelimbic-infralimbic cortices in rats do not disrupt response selection processes but induce delay-dependent deficits: evidence for a role in working memory? Behav. Neurosci. 113, 941–955 10.1037/0735-7044.113.5.941 10571477

[B33] Delong M. R. Wichmann T. (2007). Circuits and circuit disorders of the basal ganglia. Arch. Neurol. 64, 20–24 10.1001/archneur.64.1.20 17210805

[B34] Denenberg V. H. (1981). Hemispheric laterality in animals and the effects of early experience. Behav. Brain Sci. 4, 1–49 10.1017/S0140525X0000733020133876

[B35] Denenberg V. H. Rosen G. D. (1983). Interhemispheric coupling coefficients: sex differences in brain neurochemistry. Am. J. Physiol. 245, R151–R153 6881372 10.1152/ajpregu.1983.245.2.R151

[B36] Deumens R. Blokland A. Prickaerts J. (2002). Modeling Parkinson's disease in rats: an evaluation of 6-OHDA lesions of the nigrostriatal pathway. Exp. Neurol. 175, 303–317 10.1006/exnr.2002.7891 12061862

[B37] Douglas R. Kellaway L. Mintz M. Van Wageningen G. (1987). The crossed nigrostriatal projection decussates in the ventral tegmental decussation. Brain Res. 418, 111–121 10.1016/0006-8993(87)90967-X 3117325

[B38] Drew K. L. Lyon R. A. Titeler M. Glick S. D. (1986). Asymmetry in D-2 binding in female rat striata. Brain Res. 363, 192–195 10.1016/0006-8993(86)90678-5 2936425

[B39] Fallon J. H. Wang C. Kim Y. Canepa N. Loughlin S. Seroogy K. (1983). Dopamine- and cholecystokinin-containing neurons of the crossed mesostriatal projection. Neurosci. Lett. 40, 233–238 10.1016/0304-3940(83)90044-7 6316210

[B40] Fass B. Butcher L. L. (1981). Evidence for a crossed nigrostriatal pathway in rats. Neurosci. Lett. 22, 109–113 10.1016/0304-3940(81)90072-0 7231804

[B41] Finch D. M. Gigg J. Tan A. M. Kosoyan O. P. (1995). Neurophysiology and neuropharmacology of projections from entorhinal cortex to striatum in the rat. Brain Res. 670, 233–247 10.1016/0006-8993(94)01279-Q 7538025

[B42] Fisher R. S. Boylan M. K. Hull C. D. Buchwald N. A. Levine M. S. (1986). Branched projections of cat sensorimotor cortex: multiple retrograde labeling via commissural corticocortical, decussated corticostriatal and undecussated corticostriatal axons. Brain Res. 384, 395–400 10.1016/0006-8993(86)91180-7 3779389

[B43] Fox N. A. Reed B. C. (2008). Cortical Asymmetry-effects of early experience on the development of cerebral asymmetry and approach-withdrawal in Handbook of Approach and Avoidance Motivation, ed Elliot A. J. (New York, NY: Psychology Press), 35–49

[B44] Fride E. Weinstock M. (1987). Increased interhemispheric coupling of the dopamine systems induced by prenatal stress. Brain Res. Bull. 18, 457–461 10.1016/0361-9230(87)90020-7 2438016

[B45] Frohna P. A. Neal-Beliveau B. S. Joyce J. N. (1997). Delayed plasticity of the mesolimbic dopamine system following neonatal 6-OHDA lesions. Synapse 25, 293–305 10.1002/(SICI)1098-2396(199703)25:3<293::AID-SYN9>3.0.CO;2-6 9068128

[B46] Gazzaniga M. S. (1995). Principles of human brain organization derived from split-brain studies. Neuron 14, 217–228 10.1016/0896-6273(95)90280-5 7857634

[B47] Gerfen C. R. (1984). The neostriatal mosaic: compartmentalization of corticostriatal input and striatonigral output systems. Nature 311, 461–464 10.1038/311461a0 6207434

[B48] Gerfen C. R. (1985). The neostriatal mosaic. I. Compartmental organization of projections from the striatum to the substantia nigra in the rat. J. Comp. Neurol. 236, 454–476 10.1002/cne.902360404 2414339

[B49] Gerfen C. R. Engber T. M. Mahan L. C. Susel Z. Chase T. N. Monsma F. J. (1990). D1 and D2 dopamine receptor-regulated gene expression of striatonigral and striatopallidal neurons. Science 250, 1429–1432 10.1126/science.2147780 2147780

[B50] Gerfen C. R. Herkenham M. Thibault J. (1987). The neostriatal mosaic: II. Patch- and matrix-directed mesostriatal dopaminergic and non-dopaminergic systems. J. Neurosci. 7, 3915–3934 2891799 10.1523/JNEUROSCI.07-12-03915.1987 PMC6569093

[B51] Gerfen C. R. Staines W. A. Arbuthnott G. W. Fibiger H. C. (1982). Crossed connections of the substantia nigra in the rat. J. Comp. Neurol. 207, 283–303 10.1002/cne.902070308 7107988

[B52] Giardino L. (1996). Right-left asymmetry of D1- and D2-receptor density is lost in the basal ganglia of old rats. Brain Res. 720, 235–238 10.1016/0006-8993(96)00144-8 8782918

[B53] Gittis A. H. Hang G. B. Ladow E. S. Shoenfeld L. R. Atallah B. V. Finkbeiner S. (2011). Rapid target-specific remodeling of fast-spiking inhibitory circuits after loss of dopamine. Neuron 71, 858–868 10.1016/j.neuron.2011.06.035 21903079 PMC3170520

[B54] Glick S. D. Carlson J. N. (1989). Regional changes in brain dopamine and serotonin metabolism induced by conditioned circling in rats: effects of water deprivation, learning and individual differences in asymmetry. Brain Res. 504, 231–237 10.1016/0006-8993(89)91362-0 2598025

[B55] Glick S. D. Carlson J. N. Baird J. L. Maisonneuve I. M. Bullock A. E. (1988). Basal and amphetamine-induced asymmetries in striatal dopamine release and metabolism: bilateral *in vivo* microdialysis in normal rats. Brain Res. 473, 161–164 10.1016/0006-8993(88)90329-0 3208119

[B56] Glick S. D. Jerussi T. P. Zimmerberg B. (1977). Behavioral and neuropharmacological correlates of nigrostriatal asymmetry in rats, in Lateralization in the Nervous System, ed Harnad S. (Oxford: Elsevier Science), 213–249 10.1016/B978-0-12-325750-5.50020-8

[B57] Glick S. D. Ross D. A. (1981). Right-sided population bias and lateralization of activity in normal rats. Brain Res. 205, 222–225 10.1016/0006-8993(81)90737-X 7193500

[B58] Glick S. D. Ross D. A. Hough L. B. (1982). Lateral asymmetry of neurotransmitters in human brain. Brain Res. 234, 53–63 10.1016/0006-8993(82)90472-3 6120746

[B59] Glick S. D. Weaver L. M. Meibach R. C. (1981). Amphetamine enhancement of reward asymmetry. Psychopharmacology (Berl.) 73, 323–327 10.1007/BF00426459 6789352

[B60] Goel V. Tierney M. Sheesley L. Bartolo A. Vartanian O. Grafman J. (2007). Hemispheric specialization in human prefrontal cortex for resolving certain and uncertain inferences. Cereb. Cortex 17, 2245–2250 10.1093/cercor/bhl132 17158186

[B61] Golden J. P. Demaro J. A. 3rd. Knoten A. Hoshi M. Pehek E. Johnson E. M. (2013). Dopamine-dependent compensation maintains motor behavior in mice with developmental ablation of dopaminergic neurons. J. Neurosci. 33, 17095–17107 10.1523/JNEUROSCI.0890-13.2013 24155314 PMC3807031

[B62] Goldman-Rakic P. S. (2011). Circuitry of primate prefrontal cortex and regulation of behavior by representational memory. Compr. Physiol. 373–417 10.1002/cphy.cp010509

[B63] Gonzalez-Hernandez T. Barroso-Chinea P. Rodriguez M. (2004). Response of the GABAergic and dopaminergic mesostriatal projections to the lesion of the contralateral dopaminergic mesostriatal pathway in the rat. Mov. Disord. 19, 1029–1042 10.1002/mds.20206 15372592

[B64] Gotts S. J. Jo H. J. Wallace G. L. Saad Z. S. Cox R. W. Martin A. (2013). Two distinct forms of functional lateralization in the human brain. Proc. Natl. Acad. Sci. U.S.A. 110, E3435–E3444 10.1073/pnas.1302581110 23959883 PMC3767540

[B65] Graveland G. A. Difiglia M. (1985). The frequency and distribution of medium-sized neurons with indented nuclei in the primate and rodent neostriatum. Brain Res. 327, 307–311 10.1016/0006-8993(85)91524-0 3986508

[B66] Gruzelier J. H. (1999). Functional neuropsychophysiological asymmetry in schizophrenia: a review and reorientation. Schizophr. Bull. 25, 91–120 10.1093/oxfordjournals.schbul.a033370 10098916

[B67] Gurney K. Prescott T. J. Redgrave P. (2001). A computational model of action selection in the basal ganglia. I. A new functional anatomy. Biol. Cybern. 84, 401–410 10.1007/PL00007984 11417052

[B68] Hietala J. Syvalahti E. Vilkman H. Vuorio K. Rakkolainen V. Bergman J. (1999). Depressive symptoms and presynaptic dopamine function in neuroleptic-naive schizophrenia. Schizophr. Res. 35, 41–50 10.1016/S0920-9964(98)00113-3 9988840

[B69] Hnasko T. S. Hjelmstad G. O. Fields H. L. Edwards R. H. (2012). Ventral tegmental area glutamate neurons: electrophysiological properties and projections. J. Neurosci. 32, 15076–15085 10.1523/JNEUROSCI.3128-12.2012 23100428 PMC3685320

[B70] Hritcu L. Ciobica A. Artenie V. (2008). Effects of right-unilateral 6-hydroxydopamine infusion-induced memory impairment and oxidative stress: relevance for Parkinson's disease. Cent. Eur. J. Biol. 3, 250–257 10.2478/s11535-008-0023-8

[B71] Hsiao M. C. Lin K. J. Liu C. Y. Schatz D. B. (2013). The interaction between dopamine transporter function, gender differences, and possible laterality in depression. Psychiatry Res. 211, 72–77 10.1016/j.pscychresns.2012.06.004 23036826

[B72] Hsiao M. C. Lin K. J. Liu C. Y. Tzen K. Y. Yen T. C. (2003). Dopamine transporter change in drug-naive schizophrenia: an imaging study with 99mTc-TRODAT-1. Schizophr. Res. 65, 39–46 10.1016/S0920-9964(03)00006-9 14623373

[B73] Ikegami M. Ichitani Y. Takahashi T. Iwasaki T. (2006). Compensatory increase in extracellular dopamine in the nucleus accumbens of adult rats with neonatal 6-hydroxydopamine treatment. Nihon Shinkei Seishin Yakurigaku Zasshi 26, 111–117 16866211

[B74] Ishikawa M. Otaka M. Huang Y. H. Neumann P. A. Winters B. D. Grace A. A. (2013). Dopamine triggers heterosynaptic plasticity. J. Neurosci. 33, 6759–6765 10.1523/JNEUROSCI.4694-12.2013 23595734 PMC3664188

[B75] Iwamoto E. T. Loh H. H. Way E. L. (1976). Circling behavior in rats with 6-hydroxydopamine or electrolytic nigral lesions. Eur. J. Pharmacol. 37, 339–356 10.1016/0014-2999(76)90042-X 986305

[B76] Jaeger C. B. Joh T. H. Reis D. J. (1983). The effect of forebrain lesions in the neonatal rat: survival of midbrain dopaminergic neurons and the crossed nigrostriatal projection. J. Comp. Neurol. 218, 74–90 10.1002/cne.902180105 6886067

[B77] Jang J. Y. Jang M. Kim S. H. Um K. B. Kang Y. K. Kim H. J. (2011). Regulation of dopaminergic neuron firing by heterogeneous dopamine autoreceptors in the substantia nigra pars compacta. J. Neurochem. 116, 966–974 10.1111/j.1471-4159.2010.07107.x 21073466

[B78] Jerussi T. P. Glick S. D. (1975). Apomorphine-induced rotation in normal rats and interaction with unilateral caudate lesions. Psychopharmacologia 40, 329–334 10.1007/BF00421471 1096216

[B79] Jerussi T. P. Glick S. D. (1976). Drug-induced rotation in rats without lesions: behavioral and neurochemical indices of a normal asymmetry in nigro-striatal function. Psychopharmacology (Berl.) 47, 249–260 10.1007/BF00427609 823560

[B80] Jerussi T. P. Taylor C. A. (1982). Bilateral asymmetry in striatal dopamine metabolism: implications for pharmacotherapy of schizophrenia. Brain Res. 246, 71–75 10.1016/0006-8993(82)90143-3 6889904

[B81] Johnson S. W. North R. A. (1992). Two types of neurone in the rat ventral tegmental area and their synaptic inputs. J. Physiol. 450, 455–468 1331427 10.1113/jphysiol.1992.sp019136 PMC1176131

[B82] Joyce J. N. (1991a). Differential response of striatal dopamine and muscarinic cholinergic receptor subtypes to the loss of dopamine. I. Effects of intranigral or intracerebroventricular 6-hydroxydopamine lesions of the mesostriatal dopamine system. Exp. Neurol. 113, 261–276 10.1016/0014-4886(91)90016-6 1833219

[B83] Joyce J. N. (1991b). Differential response of striatal dopamine and muscarinic cholinergic receptor subtypes to the loss of dopamine. II. Effects of 6-hydroxydopamine or colchicine microinjections into the VTA or reserpine treatment. Exp. Neurol. 113, 277–290 10.1016/0014-4886(91)90017-7 1833220

[B84] Kalivas P. W. Churchill L. Klitenick M. A. (1993). GABA and enkephalin projection from the nucleus accumbens and ventral pallidum to the ventral tegmental area. Neuroscience 57, 1047–1060 10.1016/0306-4522(93)90048-K 7508582

[B85] Karreman M. Moghaddam B. (1996). The prefrontal cortex regulates the basal release of dopamine in the limbic striatum: an effect mediated by ventral tegmental area. J. Neurochem. 66, 589–598 10.1046/j.1471-4159.1996.66020589.x 8592128

[B86] Kelly E. Jenner P. Marsden C. D. (1984). Behavioural effects mediated by unilateral nigral dopamine receptor stimulation in the rat. Exp. Brain Res. 55, 243–252 10.1007/BF00237275 6611272

[B87] Kemp J. M. Powell T. P. (1971). The structure of the caudate nucleus of the cat: light and electron microscopy. Philos. Trans. R. Soc. Lond. B Biol. Sci. 262, 383–401 10.1098/rstb.1971.0102 4107495

[B88] Kesner R. P. Hunt M. E. Williams J. M. Long J. M. (1996). Prefrontal cortex and working memory for spatial response, spatial location, and visual object information in the rat. Cereb. Cortex 6, 311–318 10.1093/cercor/6.2.311 8670659

[B89] Kimura D. Archibald Y. (1974). Motor functions of the left hemisphere. Brain 97, 337–350 10.1093/brain/97.1.337 4434181

[B90] Kita H. (1992). Responses of globus pallidus neurons to cortical stimulation: intracellular study in the rat. Brain Res. 589, 84–90 10.1016/0006-8993(92)91164-A 1422824

[B91] Ko J. H. Monchi O. Ptito A. Bloomfield P. Houle S. Strafella A. P. (2008). Theta burst stimulation-induced inhibition of dorsolateral prefrontal cortex reveals hemispheric asymmetry in striatal dopamine release during a set-shifting task: a TMS-[(11)C]raclopride PET study. Eur. J. Neurosci. 28, 2147–2155 10.1111/j.1460-9568.2008.06501.x 19046396 PMC2967524

[B92] Kolb B. Buhrmann K. McDonald R. Sutherland R. J. (1994). Dissociation of the medial prefrontal, posterior parietal, and posterior temporal cortex for spatial navigation and recognition memory in the rat. Cereb. Cortex 4, 664–680 10.1093/cercor/4.6.664 7703691

[B93] Koob G. F. Stinus L. Le Moal M. (1981). Hyperactivity and hypoactivity produced by lesions to the mesolimbic dopamine system. Behav. Brain Res. 3, 341–359 10.1016/0166-4328(81)90004-8 6796098

[B94] Kopniczky Z. Dochnal R. Macsai M. Pal A. Kiss G. Mihaly A. (2006). Alterations of behavior and spatial learning after unilateral entorhinal ablation of rats. Life Sci. 78, 2683–2688 10.1016/j.lfs.2005.10.014 16313927

[B95] Kravitz A. V. Freeze B. S. Parker P. R. Kay K. Thwin M. T. Deisseroth K. (2010). Regulation of parkinsonian motor behaviours by optogenetic control of basal ganglia circuitry. Nature 466, 622–626 10.1038/nature09159 20613723 PMC3552484

[B96] Krayniak P. F. Meibach R. C. Siegel A. (1981). A projection from the entorhinal cortex to the nucleus accumbens in the rat. Brain Res. 209, 427–431 10.1016/0006-8993(81)90165-7 7225802

[B97] Kurachi M. Sumiyoshi T. Shibata R. Sun Y. J. Uehara T. Tanii Y. (2000). Changes in limbic dopamine metabolism following quinolinic acid lesions of the left entorhinal cortex in rats. Psychiatry Clin. Neurosci. 54, 83–89 10.1046/j.1440-1819.2000.00641.x 15558884

[B98] Laplante F. Dufresne M. M. Ouboudinar J. Ochoa-Sanchez R. Sullivan R. M. (2013). Reduction in cholinergic interneuron density in the nucleus accumbens attenuates local extracellular dopamine release in response to stress or amphetamine. Synapse 67, 21–29 10.1002/syn.21612 23034725

[B99] Lawler C. P. Gilmore J. H. Watts V. J. Walker Q. D. Southerland S. B. Cook L. L. (1995). Interhemispheric modulation of dopamine receptor interactions in unilateral 6-OHDA rodent model. Synapse 21, 299–311 10.1002/syn.890210404 8869160

[B100] Lieu C. A. Subramanian T. (2012). The interhemispheric connections of the striatum: implications for Parkinson's disease and drug-induced dyskinesias. Brain Res. Bull. 87, 1–9 10.1016/j.brainresbull.2011.09.013 21963946 PMC3246032

[B101] Lisman J. E. Grace A. A. (2005). The hippocampal-VTA loop: controlling the entry of information into long-term memory. Neuron 46, 703–713 10.1016/j.neuron.2005.05.002 15924857

[B102] Loughlin S. E. Fallon J. H. (1982). Mesostriatal projections from ventral tegmentum and dorsal raphe: cells project ipsilaterally or contralaterally but not bilaterally. Neurosci. Lett. 32, 11–16 10.1016/0304-3940(82)90221-X 6183620

[B103] Loughlin S. E. Fallon J. H. (1984). Substantia nigra and ventral tegmental area projections to cortex: topography and collateralization. Neuroscience 11, 425–435 10.1016/0306-4522(84)90034-4 6201780

[B104] Louilot A. Choulli M. K. (1997). Asymmetrical increases in dopamine turn-over in the nucleus accumbens and lack of changes in locomotor responses following unilateral dopaminergic depletions in the entorhinal cortex. Brain Res. 778, 150–157 10.1016/S0006-8993(97)01050-0 9462887

[B105] Louilot A. Le Moal M. (1994). Lateralized interdependence between limbicotemporal and ventrostriatal dopaminergic transmission. Neuroscience 59, 495–500 10.1016/0306-4522(94)90171-6 7911983

[B106] Lupinsky D. Moquin L. Gratton A. (2010). Interhemispheric regulation of the medial prefrontal cortical glutamate stress response in rats. J. Neurosci. 30, 7624–7633 10.1523/JNEUROSCI.1187-10.2010 20519537 PMC6632388

[B107] Martin-Soelch C. Szczepanik J. Nugent A. Barhaghi K. Rallis D. Herscovitch P. (2011). Lateralization and gender differences in the dopaminergic response to unpredictable reward in the human ventral striatum. Eur. J. Neurosci. 33, 1706–1715 10.1111/j.1460-9568.2011.07642.x 21453423 PMC3086965

[B109] McGeorge A. J. Faull R. L. (1989). The organization of the projection from the cerebral cortex to the striatum in the rat. Neuroscience 29, 503–537 10.1016/0306-4522(89)90128-0 2472578

[B110] Mensah P. Deadwyler S. (1974). The caudate nucleus of the rat: cell types and the demonstration of a commissural system. J. Anat. 117, 281–293 4142635 PMC1231402

[B111] Meshul C. K. Allen C. (2000). Haloperidol reverses the changes in striatal glutamatergic immunolabeling following a 6-OHDA lesion. Synapse 36, 129–142 10.1002/(SICI)1098-2396(200005)36:2<129::AID-SYN6>3.0.CO;2-4 10767060

[B112] Meshul C. K. Emre N. Nakamura C. M. Allen C. Donohue M. K. Buckman J. F. (1999). Time-dependent changes in striatal glutamate synapses following a 6-hydroxydopamine lesion. Neuroscience 88, 1–16 10.1016/S0306-4522(98)00189-4 10051185

[B113] Mogenson G. J. Jones D. L. Yim C. Y. (1980). From motivation to action: functional interface between the limbic system and the motor system. Prog. Neurobiol. 14, 69–97 10.1016/0301-0082(80)90018-0 6999537

[B114] Mohr C. Bracha H. S. (2004). Compound measure of hand-foot-eye preference masked opposite turning behavior in healthy right-handers and non-right-handers: technical comment on Mohr et al. (2003). Behav. Neurosci. 118, 1145–1146 10.1037/0735-7044.118.5.1145 15506901

[B115] Mohr C. Landis T. Bracha H. S. Brugger P. (2003). Opposite turning behavior in right-handers and non-right-handers suggests a link between handedness and cerebral dopamine asymmetries. Behav. Neurosci. 117, 1448–1452 10.1037/0735-7044.117.6.1448 14674863

[B118] Moser E. I. Witter M. P. Moser M. B. (2010). Entorhinal cortex in Handbook of Brain Microcircuits, eds Gordon S. M. Grillner S. (New York, NY: Oxford University Press), 175–192

[B119] Nambu A. Tokuno H. Hamada I. Kita H. Imanishi M. Akazawa T. (2000). Excitatory cortical inputs to pallidal neurons via the subthalamic nucleus in the monkey. J. Neurophysiol. 84, 289–300 10899204 10.1152/jn.2000.84.1.289

[B120] Nambu A. Tokuno H. Takada M. (2002). Functional significance of the cortico-subthalamo-pallidal ‘hyperdirect’ pathway. Neurosci. Res. 43, 111–117 10.1016/S0168-0102(02)00027-5 12067746

[B121] Nauta W. J. Smith G. P. Faull R. L. Domesick V. B. (1978). Efferent connections and nigral afferents of the nucleus accumbens septi in the rat. Neuroscience 3, 385–401 10.1016/0306-4522(78)90041-6 683502

[B122] Nielsen D. M. Visker K. E. Cunningham M. J. Keller R. W. Jr. Glick S. D. Carlson J. N. (1997). Paw preference, rotation, and dopamine function in Collins HI and LO mouse strains. Physiol. Behav. 61, 525–535 10.1016/S0031-9384(96)00496-9 9108571

[B123] Nowak G. (1989). Lateralization of neocortical dopamine receptors and dopamine level in normal Wistar rats. Pol. J. Pharmacol. Pharm. 41, 133–137 2687822

[B124] Oertel W. H. Mugnaini E. (1984). Immunocytochemical studies of GABAergic neurons in rat basal ganglia and their relations to other neuronal systems. Neurosci. Lett. 47, 233–238 10.1016/0304-3940(84)90519-6 6147799

[B126] Olpe H. R. Schellenberg H. Koella W. P. (1977). Rotational behavior induced in rats by intranigral application of GABA-related drugs and GABA antagonists. Eur. J. Pharmacol. 45, 291–294 10.1016/0014-2999(77)90012-7 562761

[B127] Ossowska K. Wolfarth S. (1995). Stimulation of glutamate receptors in the intermediate/caudal striatum induces contralateral turning. Eur. J. Pharmacol. 273, 89–97 10.1016/0014-2999(94)00671-S 7737321

[B128] Parent A. Hazrati L. N. (1995). Functional anatomy of the basal ganglia. I. The cortico-basal ganglia-thalamo-cortical loop. Brain Res. Brain Res. Rev. 20, 91–127 10.1016/0165-0173(94)00007-C 7711769

[B129] Peterson B. S. Riddle M. A. Cohen D. J. Katz L. D. Smith J. C. Leckman J. F. (1993). Human basal ganglia volume asymmetries on magnetic resonance images. Magn. Reson. Imaging 11, 493–498 10.1016/0730-725X(93)90468-S 8316062

[B130] Phillipson O. T. (1979). Afferent projections to the ventral tegmental area of Tsai and interfascicular nucleus: a horseradish peroxidase study in the rat. J. Comp. Neurol. 187, 117–143 10.1002/cne.901870108 489776

[B131] Pritzel M. Huston J. P. Sarter M. (1983). Behavioral and neuronal reorganization after unilateral substantia nigra lesions: evidence for increased interhemispheric nigrostriatal projections. Neuroscience 9, 879–888 10.1016/0306-4522(83)90276-2 6413887

[B132] Rodriguez M. Martin L. Santana C. (1994). Ontogenic development of brain asymmetry in dopaminergic neurons. Brain Res. Bull. 33, 163–171 10.1016/0361-9230(94)90246-1 8275334

[B133] Roedter A. Winkler C. Samii M. Walter G. F. Brandis A. Nikkhah G. (2001). Comparison of unilateral and bilateral intrastriatal 6-hydroxydopamine-induced axon terminal lesions: evidence for interhemispheric functional coupling of the two nigrostriatal pathways. J. Comp. Neurol. 432, 217–229 10.1002/cne.1098 11241387

[B135] Scheel-Kruger J. Arnt J. Magelund G. (1977). Behavioural stimulation induced by muscimol and other GABA agonists injected into the substantia nigra. Neurosci. Lett. 4, 351–356 10.1016/0304-3940(77)90183-5 19556189

[B136] Schneider L. H. Murphy R. B. Coons E. E. (1982). Lateralization of striatal dopamine (D2) receptors in normal rats. Neurosci. Lett. 33, 281–284 10.1016/0304-3940(82)90385-8 7162690

[B137] Seibyl J. P. Marek K. L. Quinlan D. Sheff K. Zoghbi S. Zea-Ponce Y. (1995). Decreased single-photon emission computed tomographic [123I]beta-CIT striatal uptake correlates with symptom severity in Parkinson's disease. Ann. Neurol. 38, 589–598 10.1002/ana.410380407 7574455

[B138] Silva M. A. Topic B. Lamounier-Zepter V. Huston J. P. Tomaz C. Barros M. (2007). Evidence for hemispheric specialization in the marmoset (*Callithrix penicillata*) based on lateralization of behavioral/neurochemical correlations. Brain Res. Bull. 74, 416–428 10.1016/j.brainresbull.2007.07.012 17920450

[B139] Simola N. Morelli M. Carta A. R. (2007). The 6-hydroxydopamine model of Parkinson's disease. Neurotox. Res. 11, 151–167 10.1007/BF03033565 17449457

[B140] Smith A. D. Amalric M. Koob G. F. Zigmond M. J. (2002). Effect of bilateral 6-hydroxydopamine lesions of the medial forebrain bundle on reaction time. Neuropsychopharmacology 26, 756–764 10.1016/S0893-133X(01)00420-1 12007746

[B142] Solstad T. Boccara C. N. Kropff E. Moser M. B. Moser E. I. (2008). Representation of geometric borders in the entorhinal cortex. Science 322, 1865–1868 10.1126/science.1166466 19095945

[B143] Stuber G. D. Britt J. P. Bonci A. (2012). Optogenetic modulation of neural circuits that underlie reward seeking. Biol. Psychiatry 71, 1061–1067 10.1016/j.biopsych.2011.11.010 22196983 PMC3332148

[B144] Sullivan R. M. (2004). Hemispheric asymmetry in stress processing in rat prefrontal cortex and the role of mesocortical dopamine. Stress 7, 131–143 10.1080/102538900410001679310 15512858

[B145] Sullivan R. M. Dufresne M. M. (2006). Mesocortical dopamine and HPA axis regulation: role of laterality and early environment. Brain Res. 1076, 49–59 10.1016/j.brainres.2005.12.100 16483551

[B146] Sullivan R. M. Gratton A. (1998). Relationships between stress-induced increases in medial prefrontal cortical dopamine and plasma corticosterone levels in rats: role of cerebral laterality. Neuroscience 83, 81–91 10.1016/S0306-4522(97)00370-9 9466400

[B147] Sullivan R. M. Szechtman H. (1995). Asymmetrical influence of mesocortical dopamine depletion on stress ulcer development and subcortical dopamine systems in rats: implications for psychopathology. Neuroscience 65, 757–766 10.1016/0306-4522(94)00531-9 7609874

[B148] Sumiyoshi T. Tsunoda M. Uehara T. Tanaka K. Itoh H. Sumiyoshi C. (2004). Enhanced locomotor activity in rats with excitotoxic lesions of the entorhinal cortex, a neurodevelopmental animal model of schizophrenia: behavioral and *in vivo* microdialysis studies. Neurosci. Lett. 364, 124–129 10.1016/j.neulet.2004.04.027 15196692

[B149] Swanson L. W. (1982). The projections of the ventral tegmental area and adjacent regions: a combined fluorescent retrograde tracer and immunofluorescence study in the rat. Brain Res. Bull. 9, 321–353 10.1016/0361-9230(82)90145-9 6816390

[B150] Szostak C. Jakubovic A. Phillips A. G. Fibiger H. C. (1986). Bilateral augmentation of dopaminergic and serotonergic activity in the striatum and nucleus accumbens induced by conditioned circling. J. Neurosci. 6, 2037–2044 3734874 10.1523/JNEUROSCI.06-07-02037.1986 PMC6568584

[B151] Szostak C. Jakubovic A. Phillips A. G. Fibiger H. C. (1989). Neurochemical correlates of conditioned circling within localized regions of the striatum. Exp. Brain Res. 75, 430–440 10.1007/BF00247949 2721620

[B152] Szostak C. Porter L. Jakubovic A. Phillips A. G. Fibiger H. C. (1988). Conditioned circling in rats: bilateral involvement of the mesotelencephalic dopamine system demonstrated following unilateral 6-hydroxydopamine lesions. Neuroscience 26, 395–401 10.1016/0306-4522(88)90157-1 3140048

[B153] Taber M. T. Fibiger H. C. (1995). Electrical stimulation of the prefrontal cortex increases dopamine release in the nucleus accumbens of the rat: modulation by metabotropic glutamate receptors. J. Neurosci. 15, 3896–3904 7751954 10.1523/JNEUROSCI.15-05-03896.1995 PMC6578236

[B154] Thiel C. M. Schwarting R. K. (2001). Dopaminergic lateralisation in the forebrain: relations to behavioural asymmetries and anxiety in male Wistar rats. Neuropsychobiology 43, 192–199 10.1159/000054889 11287799

[B155] Thierry A. M. Tassin J. P. Blanc G. Glowinski J. (1976). Selective activation of mesocortical DA system by stress. Nature 263, 242–244 10.1038/263242a0 958479

[B156] Tomer R. Goldstein R. Z. Wang G. J. Wong C. Volkow N. D. (2008). Incentive motivation is associated with striatal dopamine asymmetry. Biol. Psychol. 77, 98–101 10.1016/j.biopsycho.2007.08.001 17868972 PMC2413324

[B157] Tomer R. Slagter H. A. Christian B. T. Fox A. S. King C. R. Murali D. (2014). Love to win or hate to lose? Asymmetry of dopamine D2 receptor binding predicts sensitivity to reward versus punishment. J. Cogn. Neurosci. 26, 1039–1048 10.1162/jocn_a_00544 24345165 PMC3972269

[B158] Torres E. M. Dunnett S. B. (2012). 6-OHDA lesion models of Parkinson's disease in the rat in Animal Models of Movement Disorders, Vol. 2, eds Lane E. L. Dunnett S. B. (New York, NY: Humana Press), 267–279 10.1007/978-1-61779-301-1

[B159] Totterdell S. Meredith G. E. (1997). Topographical organization of projections from the entorhinal cortex to the striatum of the rat. Neuroscience 78, 715–729 10.1016/S0306-4522(96)00592-1 9153653

[B160] Uehara T. Sumiyoshi T. Matsuoka T. Itoh H. Kurachi M. (2007). Effect of prefrontal cortex inactivation on behavioral and neurochemical abnormalities in rats with excitotoxic lesions of the entorhinal cortex. Synapse 61, 391–400 10.1002/syn.20383 17372984

[B161] Uehara T. Tanii Y. Sumiyoshi T. Kurachi M. (2000). Neonatal lesions of the left entorhinal cortex affect dopamine metabolism in the rat brain. Brain Res. 860, 77–86 10.1016/S0006-8993(00)01985-5 10727625

[B162] Ungerstedt U. Arbuthnott G. W. (1970). Quantitative recording of rotational behavior in rats after 6-hydroxy-dopamine lesions of the nigrostriatal dopamine system. Brain Res. 24, 485–493 10.1016/0006-8993(70)90187-3 5494536

[B163] Van Der Kooy D. Coscina D. V. Hattori T. (1981). Is there a non-dopaminergic nigrostriatal pathway? Neuroscience 6, 345–357 10.1016/0306-4522(81)90128-7 7012667

[B164] Van Der Kooy D. Wise R. A. (1980). Retrograde fluorescent tracing of substantia nigra neurons combined with catecholamine histofluorescence. Brain Res. 183, 447–452 10.1016/0006-8993(80)90479-5 7353149

[B165] Van Dyck C. H. Seibyl J. P. Malison R. T. Laruelle M. Zoghbi S. S. Baldwin R. M. (2002). Age-related decline in dopamine transporters: analysis of striatal subregions, nonlinear effects, and hemispheric asymmetries. Am. J. Geriatr. Psychiatry 10, 36–43 10.1097/00019442-200201000-00005 11790633

[B166] Van Zessen R. Phillips J. L. Budygin E. A. Stuber G. D. (2012). Activation of VTA GABA neurons disrupts reward consumption. Neuron 73, 1184–1194 10.1016/j.neuron.2012.02.016 22445345 PMC3314244

[B167] Veening J. G. Cornelissen F. M. Lieven P. A. (1980). The topical organization of the afferents to the caudatoputamen of the rat. A horseradish peroxidase study. Neuroscience 5, 1253–1268 10.1016/0306-4522(80)90198-0 7402468

[B168] Vernaleken I. Weibrich C. Siessmeier T. Buchholz H. G. Rosch F. Heinz A. (2007). Asymmetry in dopamine D(2/3) receptors of caudate nucleus is lost with age. Neuroimage 34, 870–878 10.1016/j.neuroimage.2006.10.013 17174574

[B169] Wichmann T. Delong M. R. (1996). Functional and pathophysiological models of the basal ganglia. Curr. Opin. Neurobiol. 6, 751–758 10.1016/S0959-4388(96)80024-9 9000030

[B170] Wilson C. J. Groves P. M. (1980). Fine structure and synaptic connections of the common spiny neuron of the rat neostriatum: a study employing intracellular inject of horseradish peroxidase. J. Comp. Neurol. 194, 599–615 10.1002/cne.901940308 7451684

[B171] Wise R. A. (2002). Brain reward circuitry: insights from unsensed incentives. Neuron 36, 229–240 10.1016/S0896-6273(02)00965-0 12383779

[B172] Wurtz R. H. Hikosaka O. (1986). Role of the basal ganglia in the initiation of saccadic eye movements. Prog. Brain Res. 64, 175–190 10.1016/S0079-6123(08)63412-3 3523602

[B173] Yamamoto B. K. Freed C. R. (1982). The trained circling rat: a model for inducing unilateral caudate dopamine metabolism. Nature 298, 467–468 10.1038/298467a0 7088191

[B174] Yamamoto B. K. Freed C. R. (1984a). Asymmetric dopamine and serotonin metabolism in nigrostriatal and limbic structures of the trained circling rat. Brain Res. 297, 115–119 10.1016/0006-8993(84)90547-X 6202362

[B175] Yamamoto B. K. Freed C. R. (1984b). Reversal of amphetamine-induced circling preference in trained circling rats. Life Sci. 34, 675–682 10.1016/0024-3205(84)90232-7 6538253

[B176] Zimmerberg B. Glick S. D. Jerussi T. P. (1974). Neurochemical correlate of a spatial preference in rats. Science 185, 623–625 10.1126/science.185.4151.623 4858234

